# Case of Aneurism of the Aorta, and Case of Imperforated Anus and Vagina

**Published:** 1804-09-01

**Authors:** John Rodman

**Affiliations:** Paisley


CASE OF ANEURISM OF THE AORTA, AND CASE OF IMPERFORATED ANUS AND VAGINA. *Communicated by* JOHN RODMAN, M.D. *of Paisley.*

IN May, 1803, an unmarried woman, ætat. 30, while apparently in perfect health, suddenly fell from her seat, and was deprived in a few moments of every symptom of life. Several means were used to restore her, but she was dead.

*Dissection Two Days after Death.*

The blood-vessels of the brain were uncommonly turgid. No other diseased appearances were observed within the cranium.

The aorta was so much dilated, that from its origin to the curvature of the arch, it measured fully five inches in diameter from right to left, and was filled with coagulable lymph hardened like a cake. The external side of this part of the vessel was greatly thickened, and adhered firmly to the sternum. The internal surface of the dorsal side was ulcerated, and at a place considerably eroded, it had burst within the pericardium, which also was much distended and filled with blood.

I was informed, after the dissection, that without any known cause a tumour appeared to the right of the sternum eighteen months ago, protruding like the face of a watch, and attended with strong pulsations. About this time she applied to a surgeon for advice, but no means were used for her recovery, and the tumour soon vanished. It

It is singular that she took little notice of it after this, and seemed to feel no further inconveniency from it. She had a good appetite, menstruated regularly, and continued very corpulent.

It was remarked, that of late she had been occasionally affected with lipothymia or smooring, as it was termed, and that while walking up hill her breathing was quicker than usual, yet within three months she had travelled twenty miles in a day with apparent ease. Her period of menstruation was about the time of her death.

This case affords an instance, in which a medical man might have been deceived more readily than in the one detailed by Dr. Moody, No. 62, of this Journal; for in the former, the tumour gradually disappeared, leaving no external marks of disease; whereas, in the latter, the tumour remained as a constant memento mori. Both patients experienced a degree of health greatly beyond what theory can lead to suppose.

Being desired to visit a female child, in September, 1803, which had not the smallest vestige of an anus or vagina, although the urethra was natural, I attempted to procure a passage from the rectum without success, and the child died.

Upon opening the body, the bladder was found to be the common receptacle for the kidneys and intestines, and the only vagina from the uterus. The rectum terminated in a kind of tube, sending out a small projection like a beak, which penetrated the back part of the bladder, nigh its neck, and opened into it obliquely downwards. The uterus was united with the fundus of the bladder, and projected very little above it. Within the bladder the os uteri and ending of the rectum were distinct, but none of the meconium had passed from the intestines.

In giving this case it may be proper to mention, that during the three days which the child lived, air was frequently observed issuing out by the urethra. Attention to this circumstance may lead, in similar cases, to a different prognosis from what might otherwise be given.

*July* 18, 1804.

